# Molecular engineering of Ni–/Co–porphyrin multilayers on reduced graphene oxide sheets as bifunctional catalysts for oxygen evolution and oxygen reduction reactions†
†Electronic supplementary information (ESI) available: Characterization of rGO/(Ni^2+^/THPP/Co^2+^/THPP)_*n*_ and other related materials; electrocatalytic performance of rGO/(Ni^2+^/THPP/Co^2+^/THPP)_*n*_ and other related materials. See DOI: 10.1039/c6sc02083f


**DOI:** 10.1039/c6sc02083f

**Published:** 2016-06-27

**Authors:** Jiqing Sun, Huajie Yin, Porun Liu, Yun Wang, Xiangdong Yao, Zhiyong Tang, Huijun Zhao

**Affiliations:** a Queensland Micro- and Nanotechnology Centre , Griffith University , Nathan , Queensland 4111 , Australia . Email: x.yao@griffith.edu.au; b Centre for Clean Environment and Energy , Griffith University , Gold Coast Campus , Queensland 4222 , Australia; c Key Laboratory of Nanosystem and Hierarchical Fabrication , National Center for Nanoscience and Technology , Beijing 100190 , P. R. China . Email: zytang@nanoctr.cn ; Fax: +86-10-62656765

## Abstract

Ni– and Co–porphyrin multilayers on reduced graphene oxide (rGO) sheets are reported as novel bifunctional catalysts for the oxygen evolution reaction (OER) and the oxygen reduction reaction (ORR). After binding with organic porphyrin molecules, the catalytically-active Ni^2+^ and Co^2+^ ions are periodically constructed onto the rGO surfaces *via* the layer-by-layer (LBL) assembly technique. The resulting catalysts exhibit good performance towards both OER and ORR, which is achieved with accurate control of the composition and thickness of the multilayer structures. This work highlights the potential for the fabrication of efficient electrocatalysts *via* molecular design.

## Abstract 2

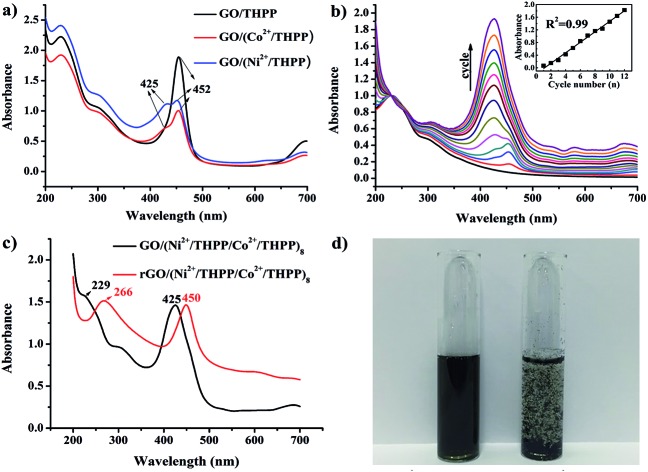
Ni– and Co–porphyrin multilayers on reduced graphene oxide (rGO) sheets are reported as novel bifunctional catalysts for the oxygen evolution reaction (OER) and the oxygen reduction reaction (ORR).

## Introduction

The shortage of fossil fuels and the daily growth of environmental pollutants call for sustainable energy solutions.^1^–^3^ Among many different ways to use energy, the hydrogen cycle is broadly deemed as the most promising conversion method due to its high energy density, renewable source and zero emission.^3^–^5^ Electrochemistry plays a key role in the hydrogen cycle, *e.g.* the conversion of electrical energy into chemical energy (water splitting) and the reverse utilization of stored chemical energy (fuel cells).^3^,^5^ Typically, water splitting involves two half reactions, named as the hydrogen evolution reaction (HER) and the oxygen evolution reaction (OER); while fuel cells experience the reverse process, called the hydrogen oxidation reaction (HOR) and the oxygen reduction reaction (ORR). It is worth pointing out that in both water splitting and fuel cells, the reactions involving oxygen (OER and ORR) are the rate-limiting processes, due to their complex four-electron process and kinetically sluggish nature.^6^,^7^ Therefore, a large overpotential is generally needed to implement both OER and ORR, leading to a low energy conversion efficiency.^8^

The search for high performance catalysts to reduce the overpotential thus becomes imperative in the electrochemistry-driven hydrogen cycle. Noble metals and noble metal oxides have been found to possess the best catalytic activities for ORR and OER; however, their scarcity, high cost and low durability render them barely economically viable.^9^–^11^ Alternatively, inspired by the effective oxygen-involving reactions in biological systems, first-row transition metal compounds have received increasing interest owing to their enzyme-like active centers, rich abundance and cheap price.^12^–^14^ Unfortunately, due to the lack of flexibility of the active centers in the rigid inorganic compounds, currently reported catalysts suffer from a rather low performance.^15^ In comparison, in biological systems, the transition metal ions are always coordinated with organic small molecules, acting as the active centers for the oxygen-involving reactions.

Herein, we suggest that the layer-by-layer (LBL) growth of Ni–/Co–porphyrin complexes on reduced graphene oxide (rGO) sheets will result in good catalyst candidates for both OER and ORR in the hydrogen cycle. The corresponding reasons are summarized as follows. (1) The structure of transition metal–porphyrin complexes is very similar to that of the active centers in enzymes.^16^–^18^ However, although high catalytic activities of transition metal–porphyrin complexes are postulated by theoretical calculations, experimental studies of transition metal–porphyrin complexes are only focused on their application in ORR^19^ and very few reports investigate OER.^20^ Obviously, a bifunctional catalyst, which can efficiently decrease the overpotential of both ORR and OER, and which is cost effective with simple operation, is highly desirable. (2) rGO has been extensively employed as a building substrate, because of its extraordinary specific surface area,^21^,^22^ high conductivity and electrochemically-inert nature. Furthermore, when the transition metal–porphyrin multilayers are adsorbed, the GO sheet substrates can immobilize the as-fabricated composites *via* strong π–π stacking and van der Waals forces.^23^–^25^ (3) The LBL growth method allows the accurate structural control of the transition metal–porphyrin complexes at the molecular level.^26^,^27^ The LBL assembly technique has been broadly recognized as a prominent method to construct functional nanostructures due to the ease of preparation, its versatility of incorporating different molecules and its precision in component arrangement.^28^–^31^ Both ORR and OER are known as complex four-electron processes coupled with proton addition or removal, and their reaction routes are generally opposite. Hence, to find a catalyst capable for effectively catalyzing both OER and ORR is a great challenge. Molecular engineering *via* LBL offers the unique opportunity to tune and balance the activity of transition metal–porphyrin catalysts involved with ORR and OER. (4) Co–/Ni–porphyrin complexes on rGO sheets possess a distinct thickness, structure and arrangement, providing an ideal model for understanding the origin of the catalytic activity.

**Scheme 1:**
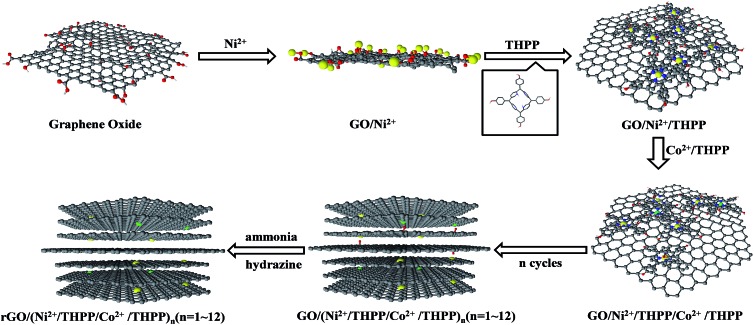
Preparation procedure of rGO/(Ni^2+^/THPP/Co^2+^/THPP)_*n*_.

Scheme 1 outlines the preparation process of Ni^2+^/Co^2+^ and 5,10,15,20-tetrakis(4-hydroxyphenyl) porphyrin (THPP) coordination complexes onto the rGO sheets *via* the LBL assembly technique. Firstly, the electrostatic attraction between Ni^2+^ and the oxygen-containing functional groups allows Ni^2+^ ions to be firmly absorbed on the GO sheet surfaces.^32^ After eliminating the excessive Ni^2+^ ions *via* centrifugation and re-dispersion in pure water, a THPP ethanol solution is introduced. Owing to the coordinate interaction between Ni^2+^ and the conjugate rings of the THPP molecules, as well as the π–π stacking and van der Waals forces between the THPP molecules and GO,^33^,^34^ the THPP molecules spontaneously absorb on the GO sheets to form a uniform layer. Subsequently, after washing with pure water several times to remove the excessive THPP molecules, Co^2+^ ions are added into the THPP/Ni^2+^ modified GO solution. These Co^2+^ ions interact with the conjugate rings of the absorbed THPP molecules and further function as linkages to the THPP molecules added next. Repeating the above process, Ni^2+^/THPP/Co^2+^/THPP is successively incorporated into the building of the multilayer structures, which are called GO/(Ni^2+^/THPP/Co^2+^/THPP)_*n*_ (*n* stands for the cycle number).^35^ Finally, in order to improve the conductivity of the products, the GO/(Ni^2+^/THPP/Co^2+^/THPP)_*n*_ samples are reduced with hydrazine in ammonia solution to obtain rGO/(Ni^2+^/THPP/Co^2+^/THPP)_*n*_.^36^

## Experimental

### Materials and instrumentation

5,10,15,20-Tetrakis(4-hydroxyphenyl) porphyrin, CoCl_2_·6H_2_O, NiCl_2_·6H_2_O, ethanol, KOH, carbon supported Ir (20 wt% Ir) (Ir/C), carbon supported Pt (20 wt% Pt) (Pt/C) and Nafion solution (5 wt%) were all purchased from the Sigma Company. All the chemicals were analytical grade (A.R.) in purity and used as received. Ultrapure water was obtained from a nanopure water system (18.2 MΩ cm, Thermo Scientific Barnstead) and used during the entire experiment.

Transmission electron microscopy (TEM) imaging and energy dispersive X-ray spectroscopy (EDX) were carried out on a FEI Tecnai G2 F20 electron microscope operated at 200 kV with the software package for automated electron tomography. Scanning electron microscopy (SEM) images were recorded with Hitachi S4800 field emission scanning electron microscope at 10 kV. X-ray photon spectroscopy (XPS) results were achieved by using a Thermo Scientific ESCALAB 250 Xi XPS system with the analysis chamber being 1.5 × 10^–9^ mbar and the size of X-ray spot being 500 μm.

### Synthesis

Graphene oxide (GO) was prepared following the typical Hummers method.^37^ Then, 50 mg GO was well dissolved into 200 mL deionized water, and 1 mL 100 mM NiCl_2_ solution was added to the GO solution. After magnetically stirring for 3 h, the obtained solution was treated by repetitive centrifugation and washing with pure water to remove excess Ni^2+^. Subsequently, the purified GO/Ni^2+^ products were re-dispersed in pure water, and 1 mL 5 mM THPP ethanol solution was added. After magnetically stirring for 12 h, the mixed solution was successively washed by water twice to get rid of the excess THPP molecules. As a result, the first layer of Ni^2+^/THPP was formed on the GO sheets. For the absorption of the Co^2+^/THPP layer, all the procedure was the same except that NiCl_2_ was substituted with 1 mL 100 mM CoCl_2_. To obtain GO/(Ni^2+^/THPP/Co^2+^/THPP)_*n*_, the aforementioned layer absorption steps were repeated *n* times. The final product was obtained when GO/(Ni^2+^/THPP/Co^2+^/THPP)_*n*_ was reduced in the presence of hydrazine hydrate and ammonia.^36^

The samples of rGO/(Ni^2+^/THPP)_*n*_ and rGO/(Co^2+^/THPP)_*n*_ were separately synthesized following the same routine except that only one type of metal ions were introduced.

### Electrochemical measurements

#### Oxygen evolution reaction

All the electrochemical studies were carried out with a standard three electrode system using a CHI 760D electrochemical workstation (CH Instruments, USA) at room temperature. A Pt wire (*φ* = 0.5 mm) worked as the counter electrode and an Ag/AgCl electrode saturated with KCl was used as the reference electrode. The reference was calibrated against and was converted to the reversible hydrogen electrode (RHE) according to the Nernst equation.^38^ The measurement was carried out by a rotating disk electrode (RDE) with a diameter of5 mm. The electrode was fully polished by alumina slurry (1.0 μm, 0.3 μm and 0.05 μm) in sequence in advance.

To prepare the catalyst ink, 1 mL catalyst solution (0.25 mg L^–1^) was added to 30 μL 5 wt% Nafion solution, which was well mixed after 30 min ultrasonication. 10 μL of the catalytic ink was then taken out to evenly drop onto the surface of the RDE followed by drying at room temperature.

All the linear sweep voltammetry (LSV) examinations on OER were recorded at a sweeping rate of 5 mV s^–1^ and rotating speed of 1600 rpm, after the tested materials were subjected to cyclic voltammetry (CV) at a sweeping rate of 5 mV s^–1^ and rotating speed of 1600 rpm for 100 cycles to get a stable CV curve. The electrolyte in use for all of the OER related assays was 1 M KOH solution.

LSV was also performed at a scanning rate of 0.1 mV s^–1^ to achieve a Tafel plot. The tested material was subjected to CV for ∼100 cycles to get a stable CV curve. Chronopotentiometry was carried out under a constant current density of 10 mA cm^–2^. All the experiments involving RDE were performed with the working electrode rotating at the speed of 1600 rpm to diminish the deviation caused by the generated oxygen bubbles.

#### Oxygen reduction reaction

Preparation of the catalytic ink for the ORR test was the same as for the OER test mentioned above. The electrolyte employed for the ORR test was 0.1 M KOH solution pre-saturated with oxygen by continuously purging with oxygen gas for 15 min. 10 μL catalytic ink was evenly dropped onto the surface of the RDE followed by drying at room temperature. The electrode was then subjected to potential cycling between –1.0 V and 0.2 V at 50 mV s^–1^ in the oxygen-saturated electrolyte till the CV curve was stable.

Koutecky–Levich plots (*J*^–1^*vs. ω*^–1/2^) were analysed at various electrode potentials. The slopes of their linear fit lines were used to calculate the electron transfer number (*n*) based on the Koutecky–Levich equation.1
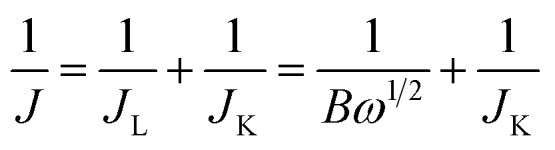

2 *B* = 0.62*nFC*_0_(*D*_0_)^2/3^*ν*^–1/6^; *J*_K_ = *nFkC*_0_

where *J* is the measured current density, *J*_K_ and *J*_L_ are the kinetic- and diffusion-limiting current densities, *ω* is the angular velocity, *n* is the transferred electron number, *F* is the Faraday constant, *C*_0_ is the bulk concentration of O_2_, *ν* is the kinematic viscosity of the electrolyte, and *k* is the electron-transfer rate constant.

#### Electrochemical impedance spectroscopy

The electrochemical impedance spectroscopy (EIS) tests were conducted with a CHI 760D electrochemical workstation under ambient conditions. For the OER, the EIS was measured at the operating potential of 1.55 V (*vs.* RHE) in 1 M KOH solution with a constant rotating speed of 1600 rpm. The impedance spectra were recorded by sweeping the frequency from 10 kHz to 1 Hz at 10 points per decade to study OER. For ORR, the EIS was measured at 0.7 V (*vs.* RHE) of each material in 0.1 M KOH solution pre-saturated with oxygen gas with the electrode stationary. The spectra were recorded by sweeping the frequency from 10 kHz to 10 Hz at 10 points per decade to investigate ORR. The obtained data were analysed *via* Zview software to get the fitting result.

## Results and discussion

**Fig. 1:**
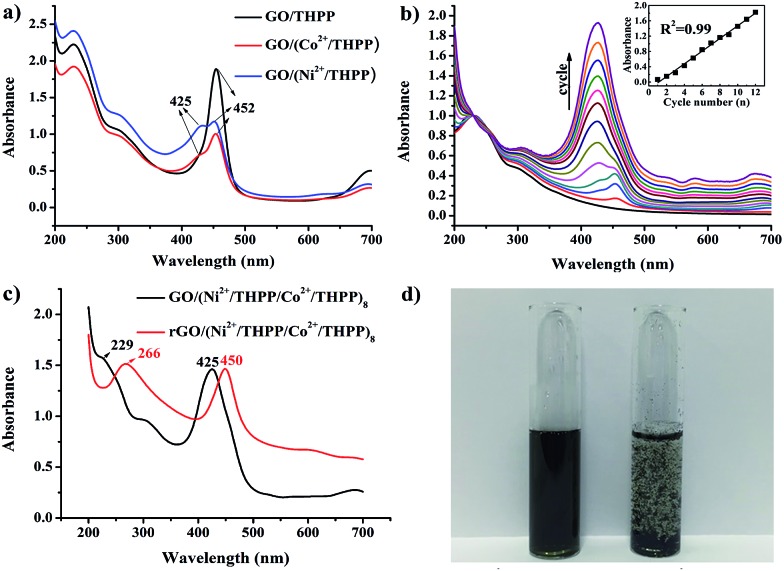
(a) UV/Vis absorption spectra of GO/THPP, GO/(Co^2+^/THPP) and GO/(Ni^2+^/THPP). (b) UV/Vis absorption spectra of rGO/(Ni^2+^/THPP/Co^2+^/THPP)_*n*_ (*n* = 2, 4, 6, 8, 10, 12). Insert: relationship of UV/Vis absorption intensity at 425 nm with increase in cycle number. (c) UV/Vis absorption spectra of GO/(Ni^2+^/THPP/Co^2+^/THPP)_8_ (before reduction) and rGO/(Ni^2+^/THPP/Co^2+^/THPP)_8_ (after reduction). (d) Solution of rGO/(Ni^2+^/THPP/Co^2+^/THPP)_8_ (left) and rGO (right) after being stored under ambient conditions for 5 months.

UV/Vis absorption spectroscopy was used to investigate the formation of GO/(Ni^2+^/THPP/Co^2+^/THPP)_*n*_ (Fig. 1). Pure THPP ethanol solution displays five characteristic absorption bands (Fig. S1†), an intense Soret band at 413 nm and four Q-bands at 516, 554, 594, and 650 nm.^30^ Upon the direct adsorption of THPP onto the GO sheets, a large red shift of the Soret band from 413 nm to 452 nm is discerned (black curve in Fig. 1a), which is ascribed to the distortion and flattening of the THPP molecules caused by their interaction with the GO sheets. The driving force for the flattening of the THPP molecules should be the electrostatic and π–π stacking interactions between GO, Ni^2+^ (Co^2+^), and the THPP molecules.^29^,^30^,^35^ As for the samples of GO/(Ni^2+^/THPP) or GO/(Co^2+^/THPP), besides the Soret band at 452 nm, a new peak appears at 425 nm (red and blue curves in Fig. 1a), showing the coordination interaction between the transition metal ions and the THPP molecules.^29^,^36^ Fig. 1b further summarizes the growth process of GO/(Ni^2+^/THPP/Co^2+^/THPP)_*n*_ monitored by UV/Vis absorption spectra after normalizing the absorption peak intensity of GO at 229 nm. It is clear that the peak intensity of GO/(Ni^2+^/THPP/Co^2+^/THPP)_*n*_ at 425 nm linearly increases with the multilayer number (insert in Fig. 1b), whereas the peak at 452 nm is gradually hidden under the peak at 425 nm. It is reasonable that the increasing amount of transition metal ions and THPP molecules on the GO surfaces screen the interaction between THPP and the GO substrate. Nevertheless, the linear increase of the peak at 425 nm highlights the gradual and uniform growth of the transition metal/THPP multilayers on the rGO substrate, offering a unique opportunity to control the amount and structure of catalysts at a molecular level.^33^,^34^,^39^,^40^ Both TEM and SEM observations give direct evidence of the multilayer uniformity (Fig. S2 and S3†), while the additional energy dispersive X-ray spectroscopy (EDX) analysis also proves the homogenous distribution of Ni, Co, N and C elements in the as-prepared composites (Fig. S4†). The conversion of GO/(Ni^2+^/THPP/Co^2+^/THPP)_*n*_ to rGO/(Ni^2+^/THPP/Co^2+^/THPP)_*n*_ is then implemented *via* a reduction reaction, and the optical properties of the products are also studied by UV/Vis absorption spectroscopy (Fig. 1c). The peak at 229 nm for GO/(Ni^2+^/THPP/Co^2+^/THPP)_8_ is red shifted to 266 nm after reduction (red curve in Fig. 1c), suggesting successful transformation of the substrate GO to rGO.^41^ Note that a simultaneous red shift is also distinguished on the THPP peak (from 425 nm to 450 nm), which can be attributed to the improved energy and electron transfer between the transition metal/THPP multilayers and the rGO substrate.^33^,^41^,^42^ Another prominent feature of rGO/(Ni^2+^/THPP/Co^2+^/THPP)_*n*_ is its excellent stability in water, which can keep well dispersed after 5 months storage (Fig. 1d). In comparison, the bare rGO sheets largely aggregate under the same storage conditions. Such good water dispersion is critical for the preparation of a uniform catalyst film on the electrode surface, and thus is beneficial for the improvement of its electrocatalytic performance.

**Fig. 2:**
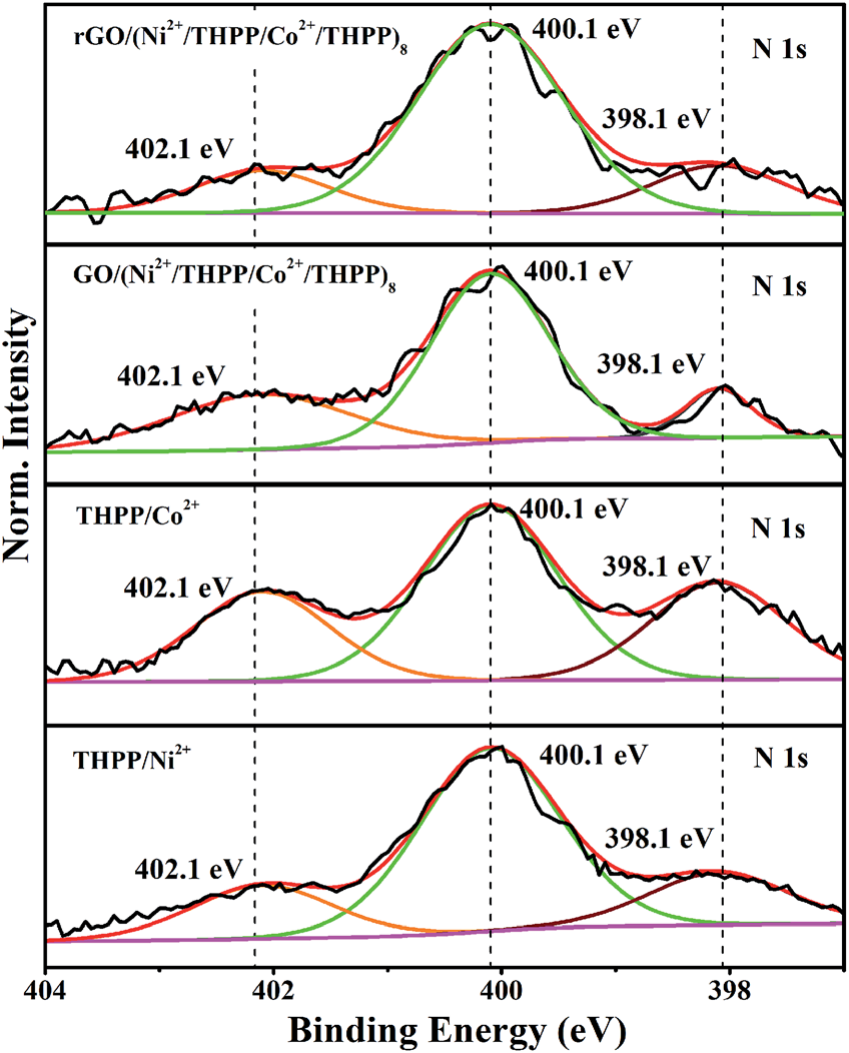
High-resolution XPS spectra of N 1s of THPP/Ni^2+^, THPP/Co^2+^, GO/(Ni^2+^/THPP/Co^2+^/THPP)_8_ and rGO/(Ni^2+^/THPP/Co^2+^/THPP)_8_.

The coordination interaction between the transition metal ions and the THPP molecules in rGO/(Ni^2+^/THPP/Co^2+^/THPP)_*n*_ was elucidated by X-ray photoelectron spectroscopy (XPS). Generally, there was only a slight change in the XPS spectra of Co 2p, Ni 2p, O 1s and N 1s before and after reduction revealing the chemical robustness of the (Ni^2+^/THPP/Co^2+^/THPP)_*n*_ multilayer films (Fig. S5† and 2). It is noteworthy that, compared with pure THPP molecules (Fig. S6†), new N 1s peaks appear at 402.1 eV for Ni^2+^/THPP, Co^2+/^THPP, GO/(Ni^2+^/THPP/Co^2+^/THPP)_8_ and rGO/(Ni^2+^/THPP/Co^2+^/THPP)_8_ (Fig. 2), demonstrating the coordination interaction of the Ni^2+^ or Co^2+^ ions with N atoms in the THPP molecules. Interestingly, the other two N 1s peaks at 398.1 eV and 400.1 eV corresponding to free N atoms in the THPP molecules still exist in both GO/(Ni^2+^/THPP/Co^2+^/THPP)_8_ and rGO/(Ni^2+^/THPP/Co^2+^/THPP)_8_, indicating the incomplete coordination between the THPP molecules and the transition metal ions in the multilayer structures.

**Fig. 3:**
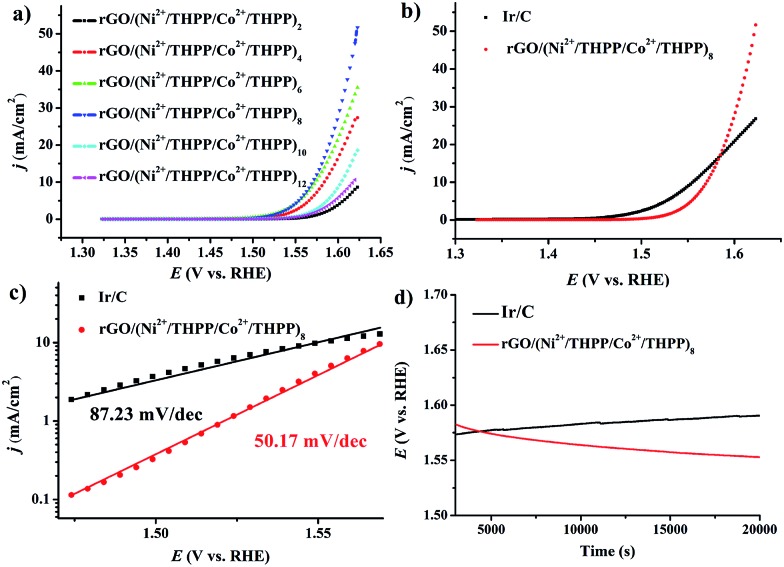
(a) LSV curves of rGO/(Ni^2+^/THPP/Co^2+^/THPP)_*n*_ (*n* = 2, 4, 6, 8, 10, 12) loaded on RDE in 1 M KOH at a sweep rate of 5 mV s^–1^ and an electrode rotating speed of 1600 rpm. (b) LSV curves of rGO/(Ni^2+^/THPP/Co^2+^/THPP)_8_ and Ir/C. (c) Tafel plot of rGO/(Ni^2+^/THPP/Co^2+^/THPP)_8_ and Ir/C. (d) Chronopotentiometry curves rGO/(Ni^2+^/THPP/Co^2+^/THPP)_8_ and Ir/C on RDE at a constant current density of 10 mA cm^–2^.

The OER activity of the different rGO/(Ni^2+^/THPP/Co^2+^/THPP)_*n*_ samples was firstly evaluated in 1 M KOH solution with a standard three-electrode system, and the uncorrected raw OER polarization curves are summarized in Fig. 3a. Evidently, the OER catalytic activity gradually increases with the multilayer number and reaches a maximum at *n* = 8, followed by a decrease with the continuous deposition of more layers. Theoretically, the catalytic activity is ameliorated with the increasing amount of the catalytic active centers.^43^ However, owing to the hydrophobic nature of the THPP molecule, the increased absorption of the THPP molecules will produce diffusion obstacles of hydrophilic OH^–^ substrates into the inner layers. The balance between more active sites and incremental hydrophobicity gives rise to the best catalytic activity of rGO/(Ni^2+^/THPP/Co^2+^/THPP)_8_. Impressively, for rGO/(Ni^2+^/THPP/Co^2+^/THPP)_8_, the onset potential is around 1.49 V *vs.* reversible hydrogen electrode (RHE), while the potential at the current density of 10 mA cm^–2^ is ∼1.56 V *vs.* RHE. Such high catalytic activity of rGO/(Ni^2+^/THPP/Co^2+^/THPP)_8_ is comparable to that of the state-of-the-art Ir/C catalyst (Fig. 3b). More interestingly, rGO/(Ni^2+^/THPP/Co^2+^/THPP)_8_ exhibits even better kinetic activity with a slope of 50.17 mV dec^–1^, (Fig. 3c), nearly half of that of the Ir/C catalyst (87.23 mV dec^–1^) (Fig. 3c). The superior kinetic activity is further highlighted by using electrochemical impedance spectroscopy (EIS) to monitor the whole OER process (Fig. S7†), which can be interpreted by the Armstrong–Henderson equivalent circuit model (Fig. S8†).^44^–^47^ As summarized in Table S1,† the variation trend of the OER charge transfer resistance (*R*_P_) is consistent with that of the OER polarization curves, in which rGO/(Ni^2+^/THPP/Co^2+^/THPP)_8_ exhibits the smallest value and thus possesses the best kinetic performance during the OER process.

The stability of rGO/(Ni^2+^/THPP/Co^2+^/THPP)_8_ was tested by chronopotentiometry. The remarkable stability of the obtained materials under the working conditions (biased galvanostatically at 10 mA cm^–2^) can be discerned from the result shown in Fig. 3d. The operating potential remains at 1.56 V and even becomes lower for up to 20 000 s. In comparison, the obvious decay of catalytic activity with increased overpotential is observed for Ir/C. The superior stability demonstrates the robust nature of the obtained catalyst, which can be assigned to the excellent elasticity and flexibility of the multilayers on the rGO substrates.

The synergistic effect between various transition metals has been reported for OER in recent years.^48^–^51^ In the transition metal/THPP multilayer systems, rGO/(Ni^2+^/THPP)_6_, rGO/(Co^2+^/THPP)_8_ and rGO/(Ni^2+^/THPP/Co^2+^/THPP)_8_ display the best catalytic activities, as demonstrated by their OER polarization curves (Fig. S9–S11†). Notably, rGO/(Ni^2+^/THPP/Co^2+^/THPP)_8_ outperforms the other two types of materials with single metal ion components (Fig. S11†), verifying the effective synergy between Ni^2+^ and Co^2+^ in the multilayers. Such a synergistic effect is further confirmed by altering the adsorption sequence of the transition metal ions, and rGO/(Ni^2+^/THPP)_4_(Co^2+^/THPP)_4_ shows an almost identical catalytic activity with respect to rGO/(Ni^2+^/THPP/Co^2+^/THPP)_8_ (Fig. S12†).

**Fig. 4:**
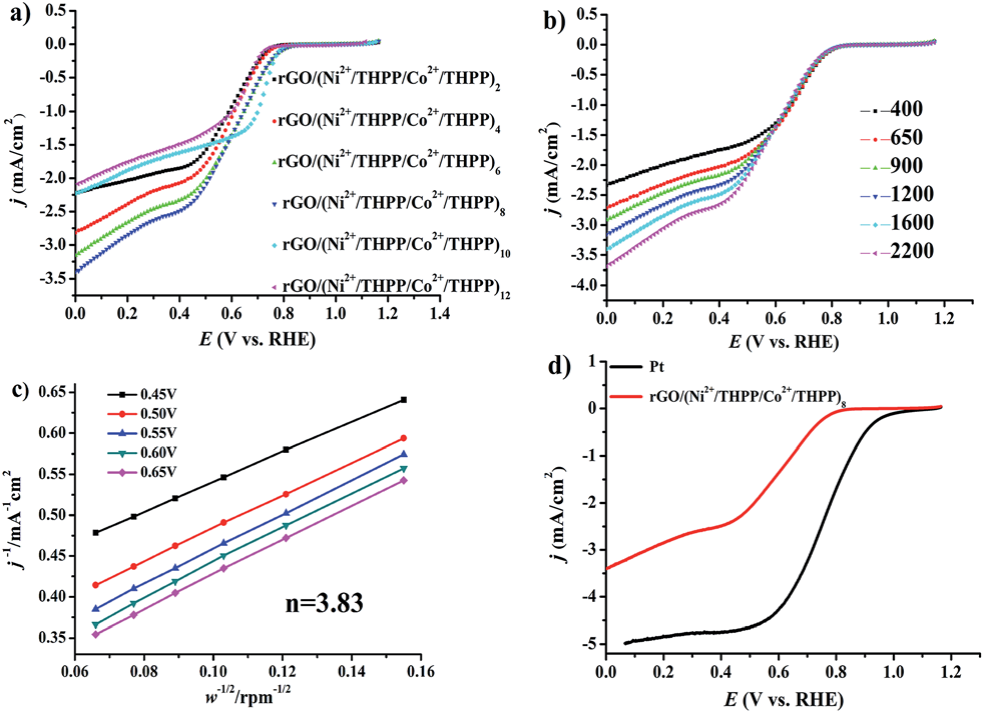
(a) LSV curves of rGO/(Ni^2+^/THPP/Co^2+^/THPP)_*n*_ (*n* = 2, 4, 6, 8, 10, 12) loaded on RDE in O_2_-saturated 0.1 M KOH at a sweep rate of 10 mV s^–1^ and an electrode rotating speed of 1600 rpm. (b) LSV on RDE of rGO/(Ni^2+^/THPP/Co^2+^/THPP)_8_ in 0.1 M KOH solution with a sweep rate of 10 mV s^–1^ at different rotating speeds. (c) Corresponding K–L plots of rGO/(Ni^2+^/THPP/Co^2+^/THPP)_8_ (*J*^–1^*vs. ω*^–0.5^) at different potentials. (d) LSV curves of rGO/(Ni^2+^/THPP/Co^2+^/THPP)_8_ and Pt/C in 0.1 M KOH at a sweep rate of 10 mV s^–1^ and an electrode rotating speed of 1600 rpm.

Subsequent evaluation of the ORR electrocatalytic activity was carried out by linear sweep voltammetry (LSV) in 0.1 M oxygen pre-saturated KOH solution under a rotating speed of 1600 rpm with a scanning rate of 10 mV s^–1^. The uncorrected raw LSV curves are outlined in Fig. 4. Analogously to OER, the obtained LSV curves of the different layers (Fig. 4a) exhibit a similar trend, namely gradual increase and subsequent decrease of the ORR catalytic activity. Impressively, the best ORR catalytic performance is also found at rGO/(Ni^2+^/THPP/Co^2+^/THPP)_8_, for which the onset potential is around 0.84 V (*vs.* RHE) and the limit current density at 0.1 V (*vs.* RHE) is around –3.3 mA cm^–2^ (Fig. 4d). EIS is also employed to kinetically monitor the whole ORR process at 0.7 V (*vs.* RHE) (Fig. S13†), and the obtained curves are then interpreted by the equivalent circuit (Fig. S14†).^52^,^53^ As listed in Table S2,† the value of reaction resistance (*R*_2_) is varied with layer number, and a consistent trend with the LSV measurement is recognized. The smallest *R*_2_ for rGO/(Ni^2+^/THPP/Co^2+^/THPP)_8_ clearly identifies that it has the best kinetic performance during the ORR process. This result also reveals that the catalytic activities of the transition metal/THPP multilayers are determined by the balance between the accumulated active centers and the increased diffusion obstacles.

To gain a deeper insight into the ORR catalytic activity, rGO/(Ni^2+^/THPP/Co^2+^/THPP)_8_ was chosen to perform the LSV curves (Fig. 4b) with a rotating disk electrode (RDE) at different rotating speeds, namely 400, 650, 900, 1200, 1600 and 2200 rpm with a sweep rate of 10 mV s^–1^. The electron-transfer number is thus calculated between 0.45 V and 0.65 V (*vs.* RHE) from the slope of the Koutecky–Levich (K–L) plots (Fig. 4c). The obtained value is around 3.83, very close to commercial Pt/C, indicating that the ORR at the rGO/(Ni^2+^/THPP/Co^2+^/THPP)_8_ electrode proceeds by an approximate four-electron reduction pathway.^40^

The possible synergistic effect between Co and Ni was also investigated for the ORR process. It is worth mentioning that, in contrast to OER, the catalytic activities of rGO/(Ni^2+^/THPP/Co^2+^/THPP)_8_, rGO/(Ni^2+^/THPP)_8_ and rGO/(Co^2+^/THPP)_6_ are rather similar, suggesting a less synergistic effect between Co and Ni for the ORR process (Fig. S15–S17†).

## Conclusions

In summary, we have successfully synthesized a robust and efficient bifunctional catalyst for OER and ORR *via* the LBL method. The synthesis process is simple, but cost-effective, environmentally-friendly and energy-saving without the demand for high temperature and sophisticated equipment. Significantly, the obtained rGO/(Ni^2+^/THPP/Co^2+^/THPP)_8_ displays excellent electrocatalytic activity towards both OER and ORR, which is comparable to state-of-the-art high performance bifunctional catalysts (Table S3†). The high controllability of the structure and catalytic activity bestowed by the molecular engineering technique not only sheds light on the rational design of atom-economic electrocatalysts, but also offers a practical way to prepare bifunctional catalysts for many important electrochemical applications including metal–air batteries, water electrolysis and fuel cells. Future efforts will be focused on shortening preparation duration, and the improving scalability of these electrocatalysts for possible practical applications.

## Supplementary Material

Supplementary information
