# Supplementary material for: Molecular engineering of Ni–/Co–porphyrin multilayers on reduced graphene oxide sheets as bifunctional catalysts for oxygen evolution and oxygen reduction reactions
Source: Chem Sci. 2016 Jun 27;7(9):5640–6. doi: 10.1039/c6sc02083f (PMC6022052; doi:10.1039/c6sc02083f)
Supplement: Supplementary file 1 [file SC-007-C6SC02083F-s001.pdf]

Electronic Supplementary Material (ESI) for Chemical Science.
This journal is © The Royal Society of Chemistry 2016

Supporting Information

Part1. Characterization of rGO/(Ni^{2+}/THPP/Co^{2+}/THPP)$_n$ and other related materials.

Part2. Electrocatalytic performance of the rGO/(Ni^{2+}/THPP/Co^{2+}/THPP)$_n$ and other related materials.

Part1. Characterization of rGO/(Ni^{2+}/THPP/Co^{2+}/THPP)$_n$ and other related materials

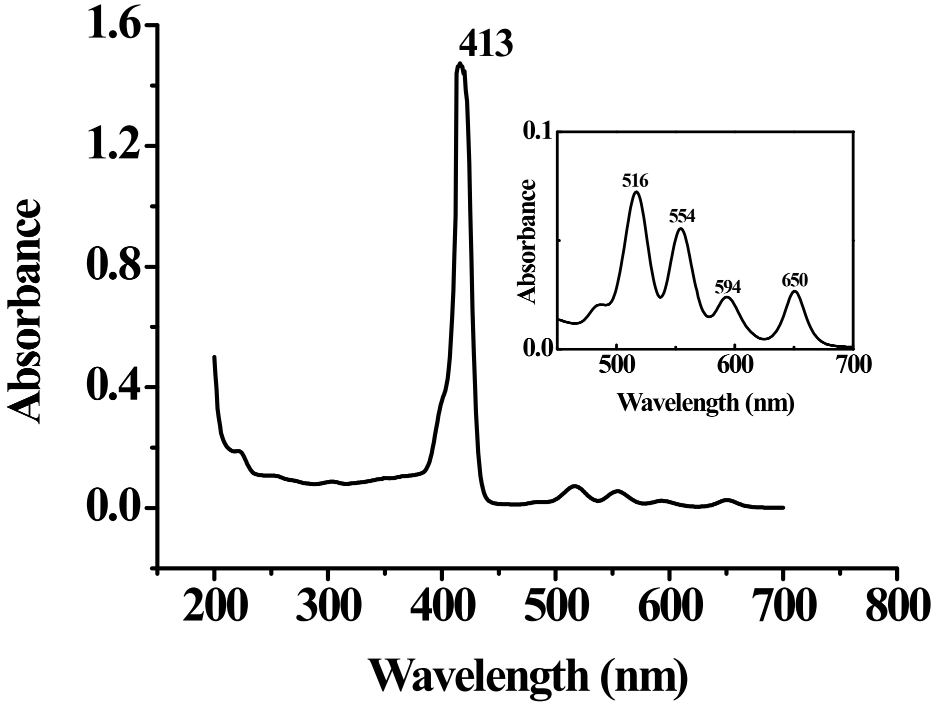

Figure S1. UV/Vis spectrum of free THPP molecules. Insert is the magnified part at wavelength from 450 nm to 700 nm.

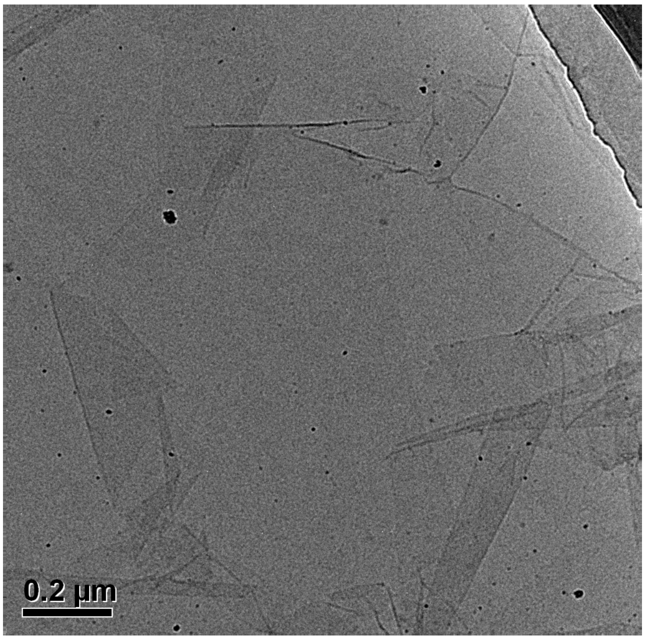

Figure S2. TEM image of rGO/(Ni^{2+}/THPP/Co^{2+}/THPP)$_8$.

Figure S2 demonstrates that (Ni^{2+}/THPP/Co^{2+}/THPP)$_8$ is homogenously grown on the rGO sheets, and no obvious aggregates are observed.

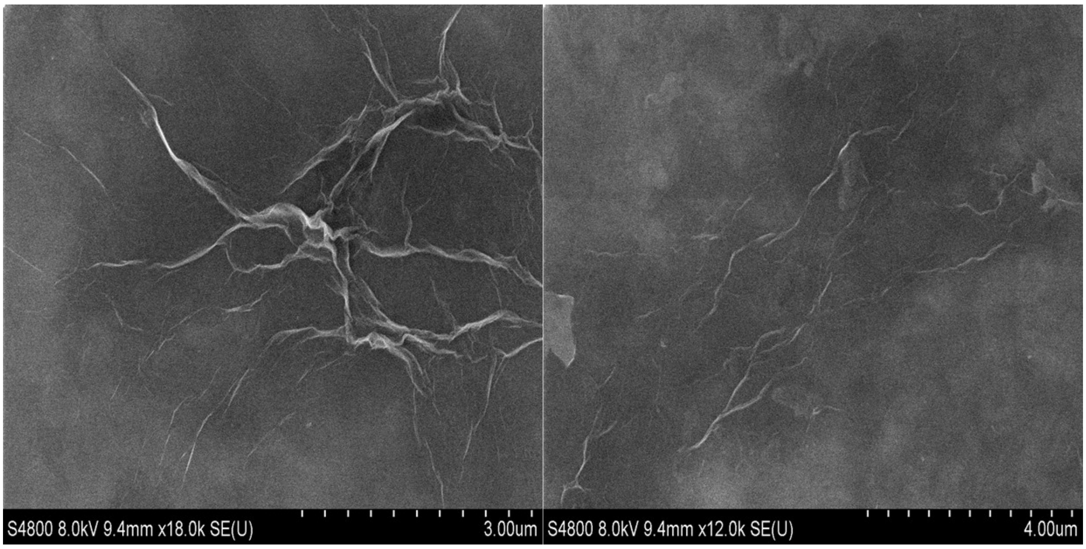

Figure S3. SEM image of rGO/(Ni^{2+}/THPP/Co^{2+}/THPP)$_8$.

Figure S3 displays the uniform growth of (Ni^{2+}/THPP/Co^{2+}/THPP)$_8$ on the rGO sheets in large scale.

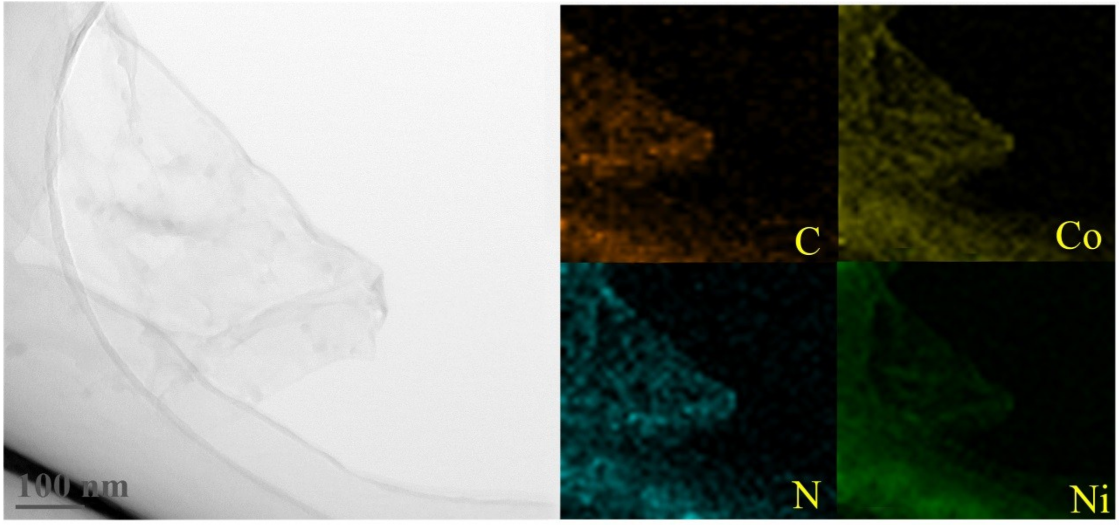

Figure S4. TEM and EDX image of rGO/(Ni^{2+}/THPP/Co^{2+}/THPP)$_8$.

The energy dispersive X-ray spectroscopy (EDX) analysis proves the homogenous distribution of Ni, Co, N and C elements in as-prepared composites.

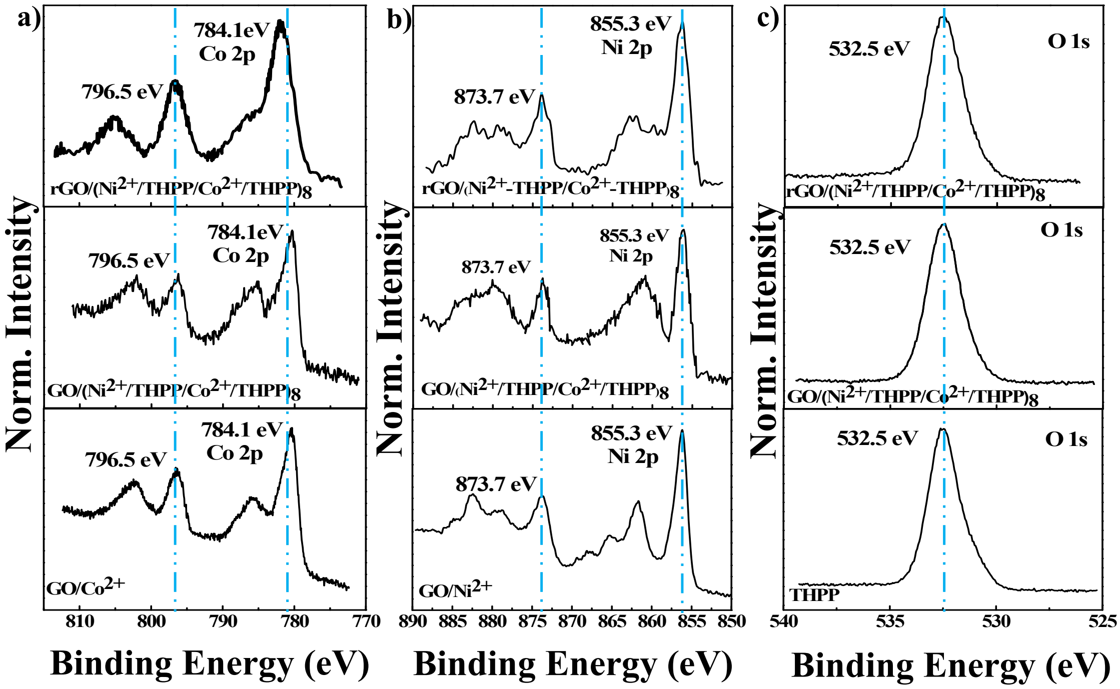

Figure S5. High-resolution XPS spectra of a) Co 2p of GO/Co^{2+}, GO/(Ni^{2+}/THPP/Co^{2+}/THPP)$_8$, and rGO/(Ni^{2+}/THPP/Co^{2+}/THPP)$_8$, b) Ni 2p of GO/Ni^{2+}, GO/(Ni^{2+}/THPP/Co^{2+}/THPP)$_8$, and rGO/(Ni^{2+}/THPP/Co^{2+}/THPP)$_8$, c) O 1s of THPP, GO/(Ni^{2+}/THPP/Co^{2+}/THPP)$_8$, and rGO/(Ni^{2+}/THPP/Co^{2+}/THPP)$_8$.

As shown in Figure S5, there is only slight change in the XPS spectra of Co 2p, Ni 2p and O 1s before and after reduction, revealing the chemical robustness of the (Ni^{2+}/THPP/Co^{2+}/THPP)$_n$ multilayer films.

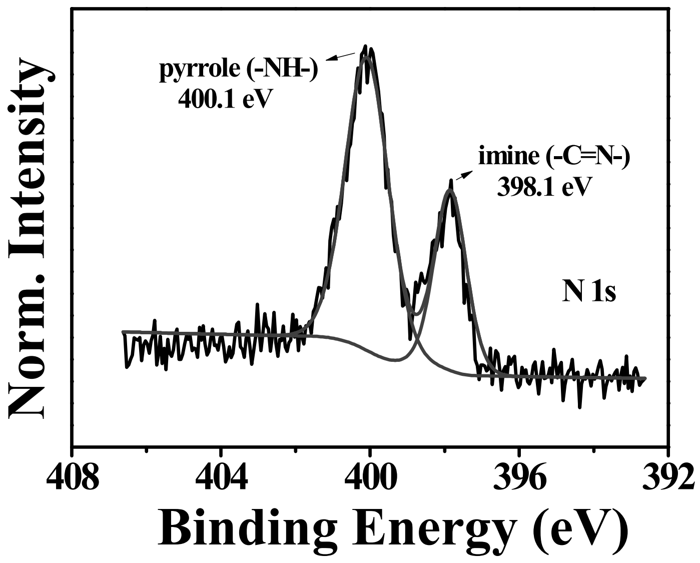

Figure S6. High resolution N 1s spectrum of free THPP molecules.

Part2. Electrocatalytic performance of rGO/(Ni^{2+}-THPP/Co^{2+}–THPP)$_n$ and other related materials

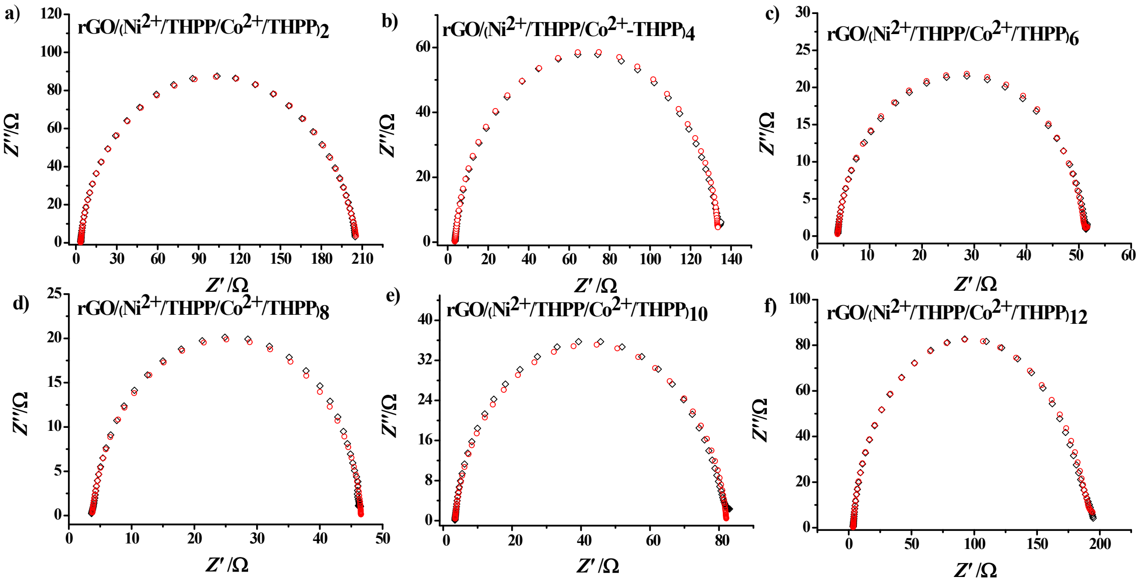

Figure S7. Nyquist plots (black diamonds) and the corresponding simulated results (red circles) of a) rGO/(Ni^{2+}/THPP/Co^{2+}/THPP)$_2$, b) rGO/(Ni^{2+}/THPP/Co^{2+}/THPP)$_4$, c) rGO/(Ni^{2+}/THPP/Co^{2+}/THPP)$_6$, d) rGO/(Ni^{2+}/THPP/Co^{2+}/THPP)$_8$, e) rGO/(Ni^{2+}/THPP/Co^{2+}/THPP)$_{10}$, and f) rGO/(Ni^{2+}/THPP/Co^{2+}/THPP)$_{12}$ at potential of 1.55V (*vs.* RHE) from 10^5 Hz - 1 Hz.

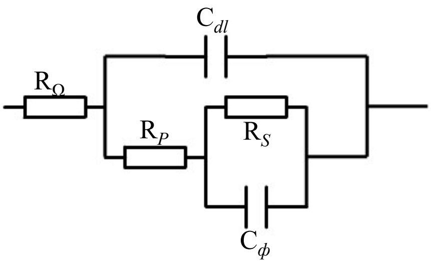

Figure S8. Armstrong-Henderson equivalent circuit model for oxygen evolution reaction.

In Figure S8, R_{Ω} represents the uncompensated solution resistance (the electrolyte resistance) and C_{dl} models the double layer capacitance associated with the catalyst/electrolyte interface. R_P denotes the OER charge transfer resistance. R_S and C_{ϕ} are the equivalent resistance and capacitance, respectively, associated with adsorption of intermediate.

Note that CPE (*constant phase element)* is introduced to replace the pure capacitance in order to fix the frequency dispersion in the capacitive response of the electrochemical system that will cause a deviation from ideal capacitive behaviour.

$$Z^{CPE} = C_{\alpha = 1}{}^{-1}(j\omega)^{-\alpha}$$

Where $C_{\alpha=1}$ is the value of the capacitance without frequency dispersion and α represents the deviation from the ideal behaviour ($\alpha \leq 1$ for a physically reasonable situation and being 1 for the perfect capacitors).

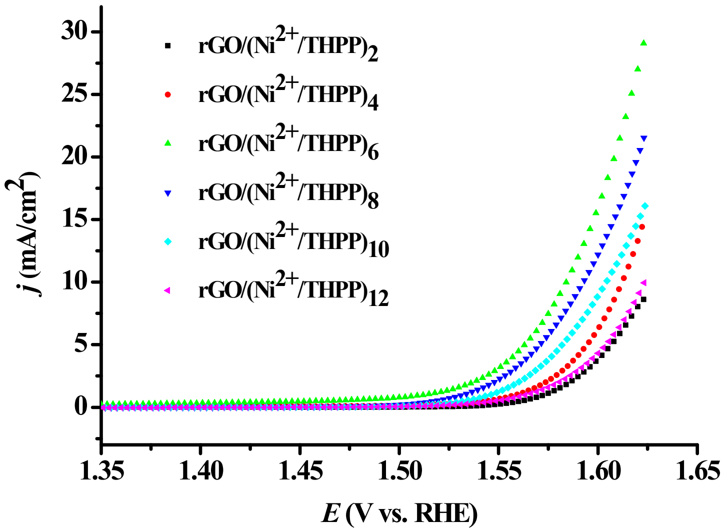

Figure S9. LSV curves of rGO/(Ni^{2+}/THPP)$_n$ (n = 2, 4, 6, 8, 10, 12) for the oxygen evolution reaction in 1 M KOH.

Figure S9 shows that rGO/(Ni^{2+}/THPP)$_6$ possesses the best OER catalytic activity among the rGO/(Ni^{2+}/THPP)$_n$ samples.

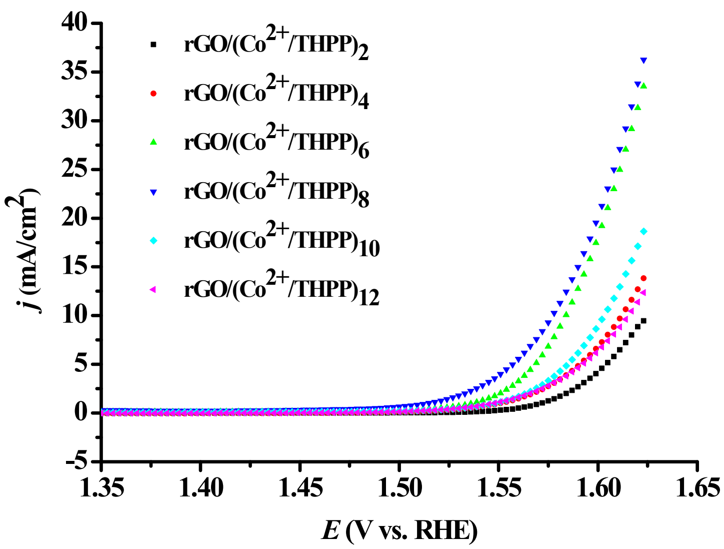

Figure S10. LSV curves of rGO/(Co^{2+}/THPP)$_n$ (n = 2, 4, 6, 8, 10, 12) for the oxygen evolution reaction in 1 M KOH.

Figure S10 shows that rGO/(Co^{2+}/THPP)$_8$ possesses the best OER catalytic activity among the rGO/(Co^{2+}/THPP)$_n$ samples.

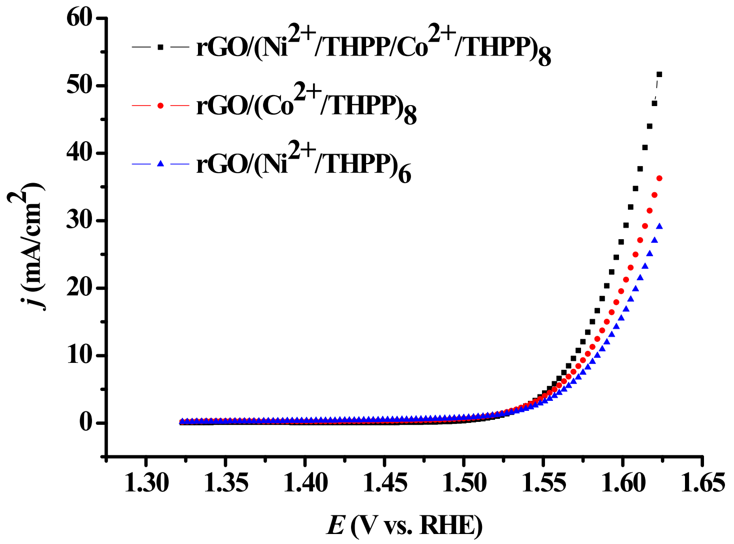

Figure S11. LSV curves of rGO/(Ni^{2+}/THPP/Co^{2+}/THPP)$_8$, rGO/(Ni^{2+}/THPP)$_6$ and rGO/(Co^{2+}/THPP)$_8$ in 1 M KOH electrolyte at rotating rate of 1600 rpm for OER.

As demonstrated in Figure S11, rGO/(Ni2+/THPP/Co2+/THPP)$_8$ outperforms the other two types of materials with single metal ion component, verifying the synergetic effect between Ni^{2+} and Co^{2+} in the mutilayers.

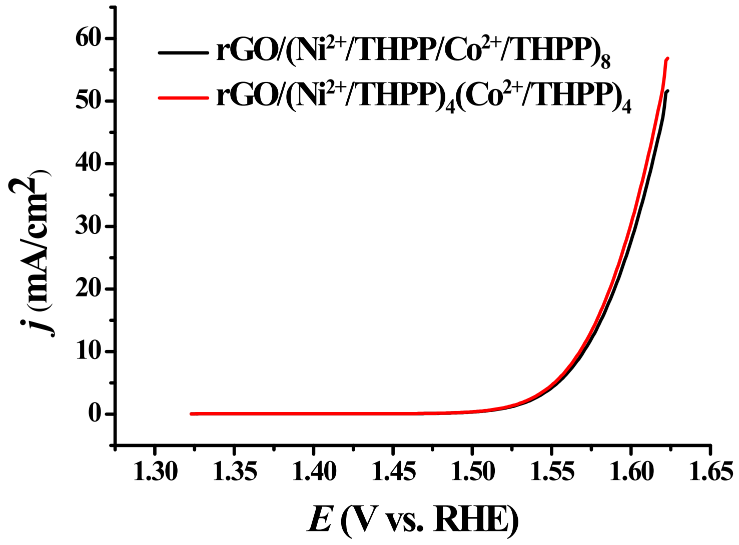

Figure S12. LSV curves of rGO/(Ni^{2+}/THPP/Co^{2+}/THPP)$_8$ and rGO/(Ni^{2+}/THPP)$_4$(Co^{2+}/THPP)$_4$ in 1 M KOH electrolyte at rotating rate of 1600 rpm for OER.

As displayed in Figure S12, rGO/(Ni^{2+}/THPP/Co^{2+}/THPP)$_8$ has the identical OER catalytic activity compared with rGO/(Ni^{2+}/THPP)$_4$(Co^{2+}/THPP)$_4$, disclosing that the adsorption sequence of the transition metal ions in the multilayers is not the key factor to determine the electrocatalytic activity.

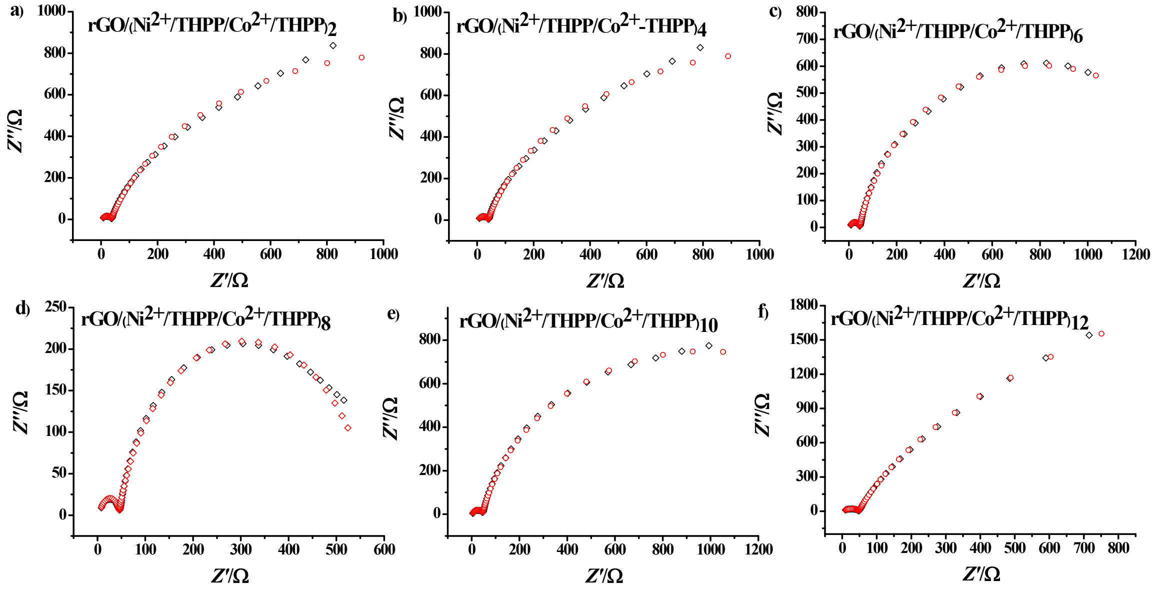

Figure S13. Nyquist plots (black diamonds) and the corresponding simulated results (red circles) of a) rGO/(Ni^{2+}/THPP/Co^{2+}/THPP)$_2$, b) rGO/(Ni^{2+}/THPP/Co^{2+}/THPP)$_4$, c) rGO/(Ni^{2+}/THPP/Co^{2+}/THPP)$_6$, d) rGO/(Ni^{2+}/THPP/Co^{2+}/THPP)$_8$, e) rGO/(Ni^{2+}/THPP/Co^{2+}/THPP)$_{10}$, and f) rGO/(Ni^{2+}/THPP/Co^{2+}/THPP)$_{12}$ at 0.7 (*vs.* RHE) from 10^5 HZ - 10Hz.

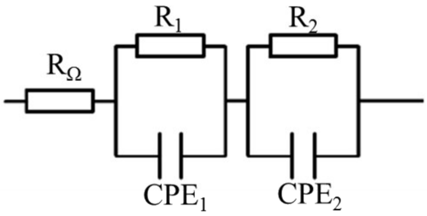

Figure S14. Equivalent circuit model for the oxygen reduction reaction.

In Figure S14, R_{Ω} represents the uncompensated solution resistance (the electrolyte resistance). R_1 and C_1 are the resistance and capacitance between the electrode and electrolyte. R_2 is the charge transfer resistance during the ORR process and C_2 denotes the double layer capacitance formed during the reaction. Note that CPE is also introduced to replace the pure capacitance to fix the frequency dispersion.

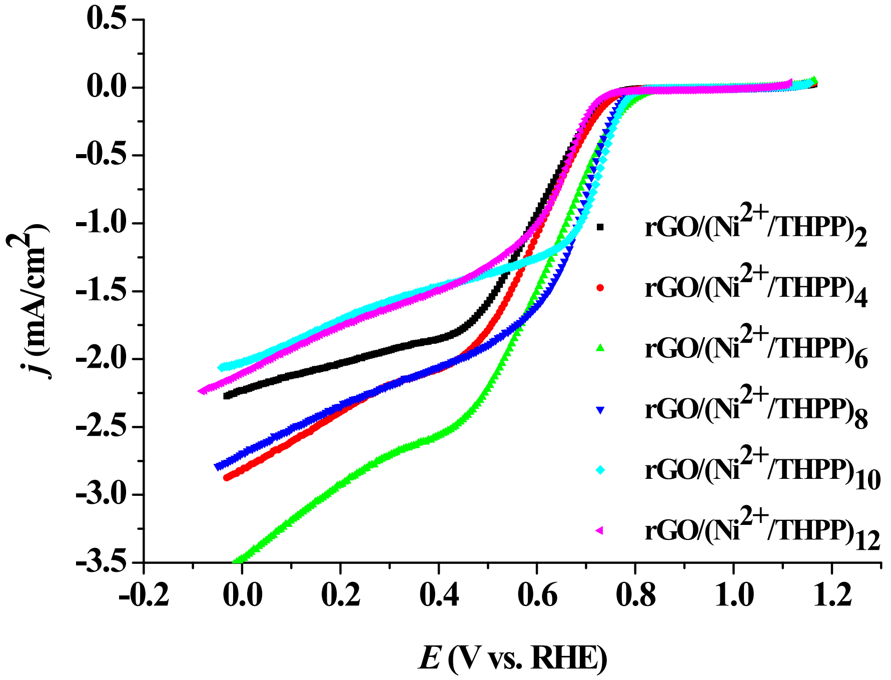

Figure S15. LSV curves of rGO/(Ni^{2+}/THPP)$_n$ (n = 2, 4, 6, 8, 10, 12) for the oxygen reduction reaction in 0.1 M KOH.

Figure S15 shows that rGO/(Ni^{2+}/THPP)$_6$ possesses the best ORR catalytic activity among the rGO/(Ni^{2+}/THPP)$_n$ samples.

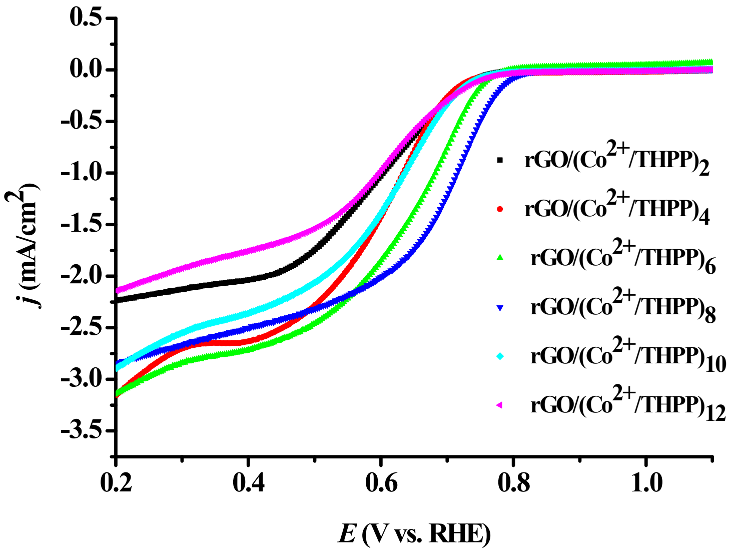

Figure S16. LSV curves of rGO/(Co^{2+}/THPP)$_n$ (n = 2, 4, 6, 8, 10, 12) for the oxygen reduction reaction in 0.1 M KOH.

Figure S16 shows that rGO/(Co^{2+}/THPP)$_8$ possesses the best OER catalytic activity among the rGO/(Co^{2+}/THPP)$_n$ samples.

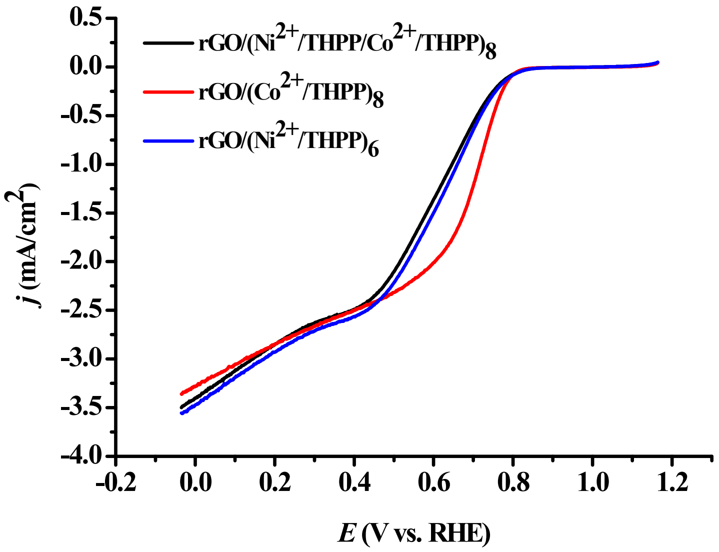

Figure S17. LSV curves of rGO/(Ni^{2+}/THPP/Co^{2+}/THPP)$_8$, rGO/(Ni^{2+}/THPP)$_6$ and rGO/(Co^{2+}/THPP)$_8$ in 0.1 M KOH electrolyte at rotating rate of 1600 rpm for ORR.

Table S1. Fitting results evaluated from the equivalent circuits of rGO/(Ni^{2+}/THPP/Co^{2+}/THPP)$_n$ (n = 2, 4 ,6, 8, 10 and 12) for OER.

n	R_{Ω}	R_P	R_S	C_{dl}	α_{dl}	C_{ϕ}	α_{ϕ}
2	3.54	187.6	14.7	1.1958E-5	0.93621	0.0011644	0.69738
4	3.63	130.7	131.8	1.5107E-5	0.92411	0.039056	0.90016
6	3.72	47.45	49.53	1.1285E-5	0.93856	0.13122	0.88555
8	3.876	11.81	31.03	9.5081E-6	0.95979	3.5246E-6	0.93861
10	3.88	61.8	16.91	1.2655E-5	0.92818	7.810E-16	0.91455
12	3.914	183.3	9.091	1.1306E-5	0.93477	0.005169	0.89314

Note that the fitted results listed in Table S1 are based on the equivalent circuits in Figure S13 and S14.

Table S2. Fitting results evaluated from the equivalent circuit of rGO/(Ni^{2+}/THPP/Co^{2+}/THPP)$_n$ (n = 2, 4, 6, 8, 10 and 12) for ORR.

n	R_Ω	R_1	C_1	α_1	R_2	C_2	α_2
2	7.282	40.14	1.1634E-8	0.9023	7092	1.1418E-5	0.88117
4	7.007	31.88	1.0939E-8	0.9046	2090	1.8956E-5	0.83702
6	6.758	37.81	1.3465E-8	0.8324	1004	1.2675E-5	0.86013
8	6.734	38.15	1.3423E-8	0.9225	529.5	1.3166E-5	0.85252
10	5.213	40.25	6.7234E-8	0.9631	1861	1.3662E-5	0.86282
12	7.171	38.26	8.5773E-9	0.9067	2137	2.0069E-4	0.81385

Note that the fitted results listed in Table S2 are based on the equivalent circuits in Figure S7 and S8.

Table S3. Performance comparison results with the state-of-art of high performance bifunctional catalysts.

Catalyst	OER onset potential (V)	OER potential at 10 mA/cm^2 (V)	ORR onset potential (V)	ORR half wave potential (V)	Δ(OER-ORR) E(V)
Co3O4[1]	1.53	1.67	0.88	0.77	0.9
PtCo[1]	1.5	1.65	0.95	0.82	0.83
NCNT/CoO-NiO-NiCo[2]	1.47	1.5	0.97	0.83	0.67
N-CG–CoO[3]	1.51	1.57	0.91	0.77	0.8
Co3O4/N-rmGO[4]	1.49	1.54	0.88	0.83	0.71
Co0.5Fe0.5S@N-MC[5]	1.57	1.64	0.913	0.808	0.73
rGO/(Ni^{2+}-THPP/Co^{2+}–THPP)$_8$ this work	1.49	1.56	0.84	0.71	0.85

[1] S. Hu, G. Goenaga, C. Melton, T. A. Zawodzinski, D. Mukherjee, *Appl. Catal. B: Environ.*, 2016, 182, 286-296.
[2] X. Liu, M. Park, M. G. Kim, S. Gupta, G. Wu, J. Cho, *Angew. Chem., Int. Ed.*,,2015, 54, 9654-9658.
[3] S. Mao, Z. Wen, T. Huang, Y. Hou, J. Chen, *Energy Environ. Sci.*, 2014, 7, 609-616.
[4] Y. Liang, Y. Li, H. Wang, J. Zhou, J. Wang, T. Regier, H. Dai, *Nature Mater.*, 2011, 10, 780-786.
[5] M. Shen, C. Ruan, Y. Chen, C. Jiang, K. Ai, L. Lu, *ACS Appl. Mater. Interfaces*, 2015, 7, 1207-1218.

As clearly manifested in Table S3, our obtained catalyst displays excellent electrocatalytic activity towards both OER and ORR, which is comparable to the state-of-art of high performance bifunctional catalysts.
